# Supplementary figures and images for: Phospholipids alter activity and stability of mitochondrial membrane-bound ubiquitin ligase MARCH5
Source: Life Sci Alliance. 2022 Apr 22;5(8):e202101309. doi: 10.26508/lsa.202101309 (PMC9034062; doi:10.26508/lsa.202101309)

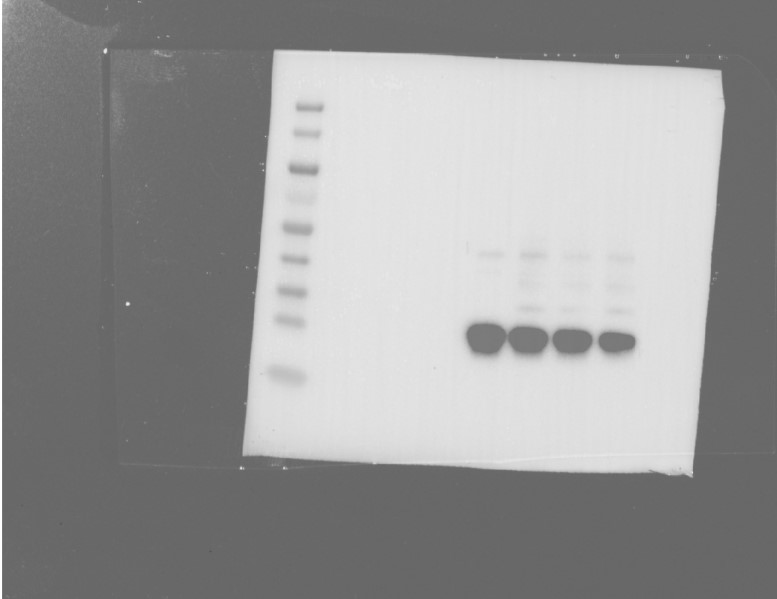

Supplement: Supplementary file 1 [file LSA-2021-01309_SdataS2.1.jpg]

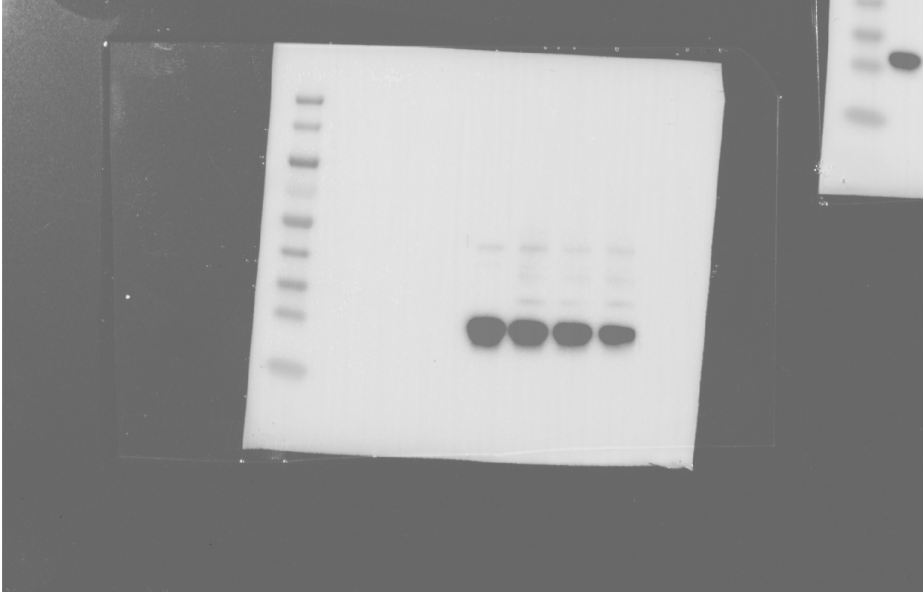

Supplement: Supplementary file 2 [file LSA-2021-01309_SdataS2.2.png]

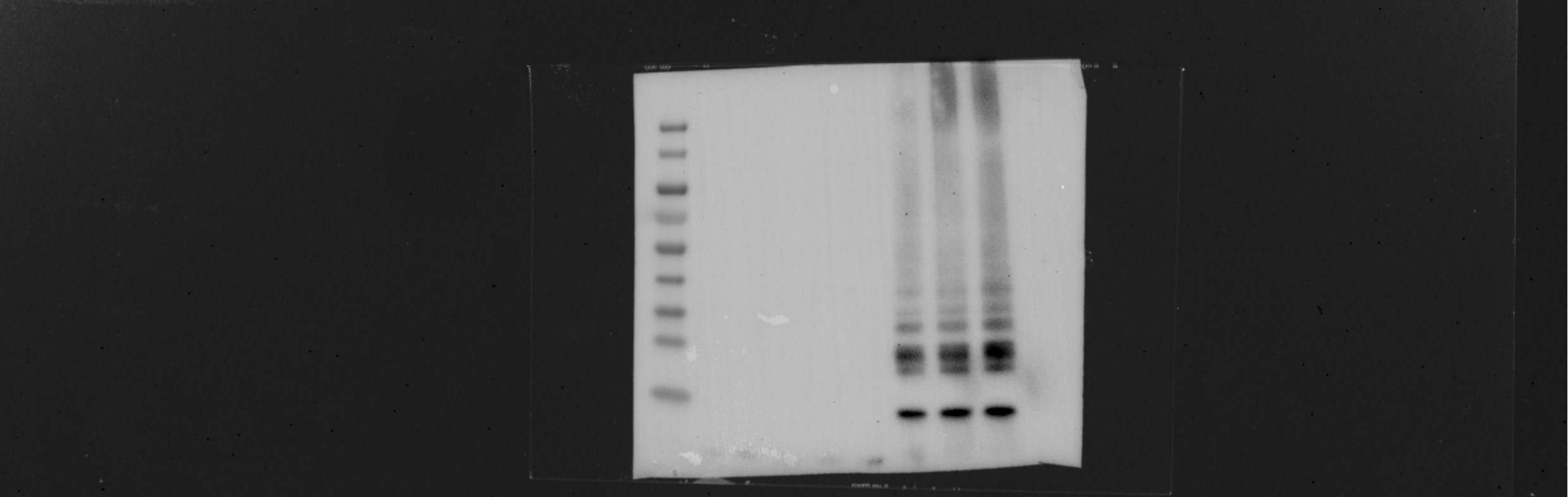

Supplement: Supplementary file 3 [file LSA-2021-01309_SdataS2.3.jpg]

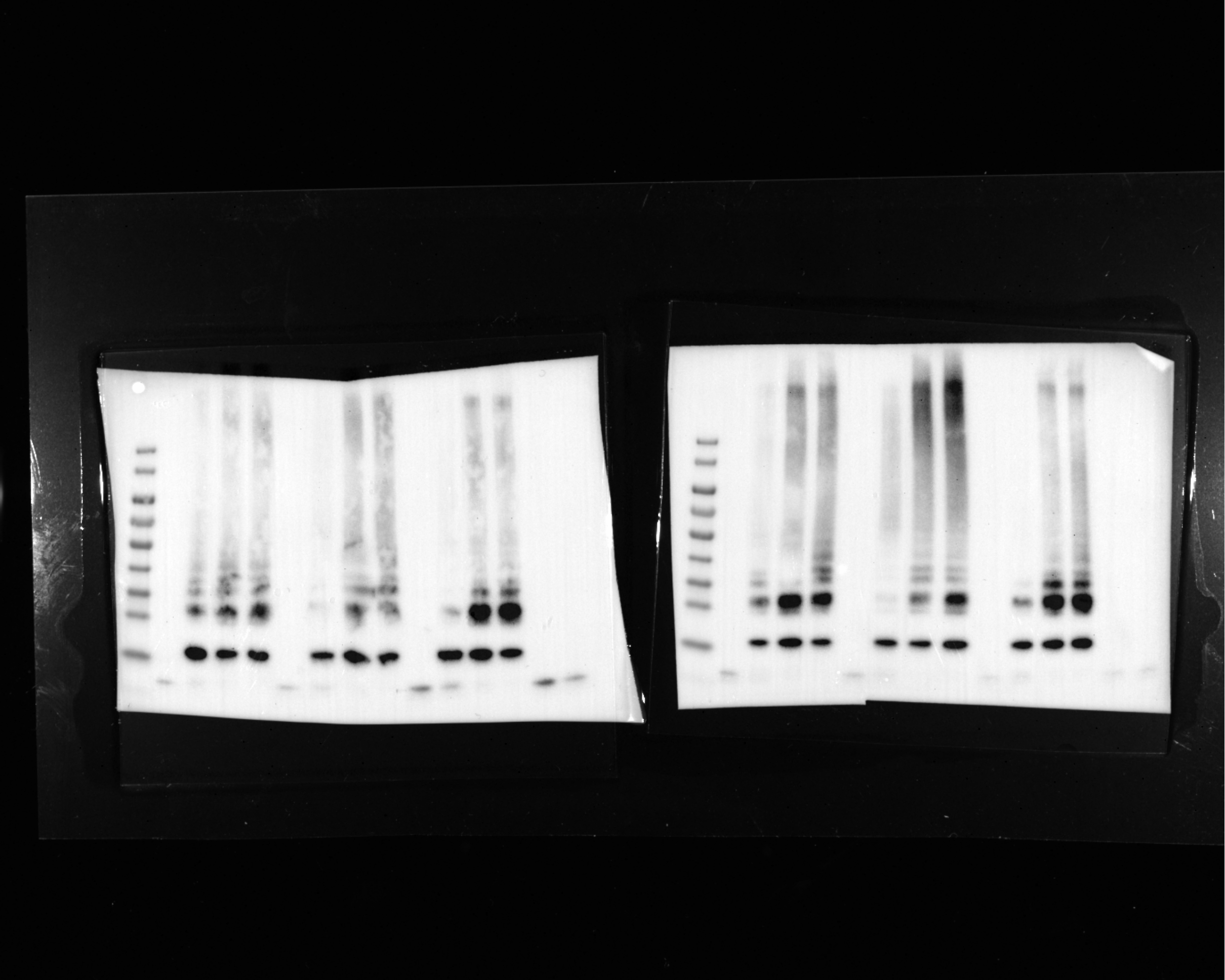

Supplement: Supplementary file 4 [file LSA-2021-01309_SdataS2.4.tif]

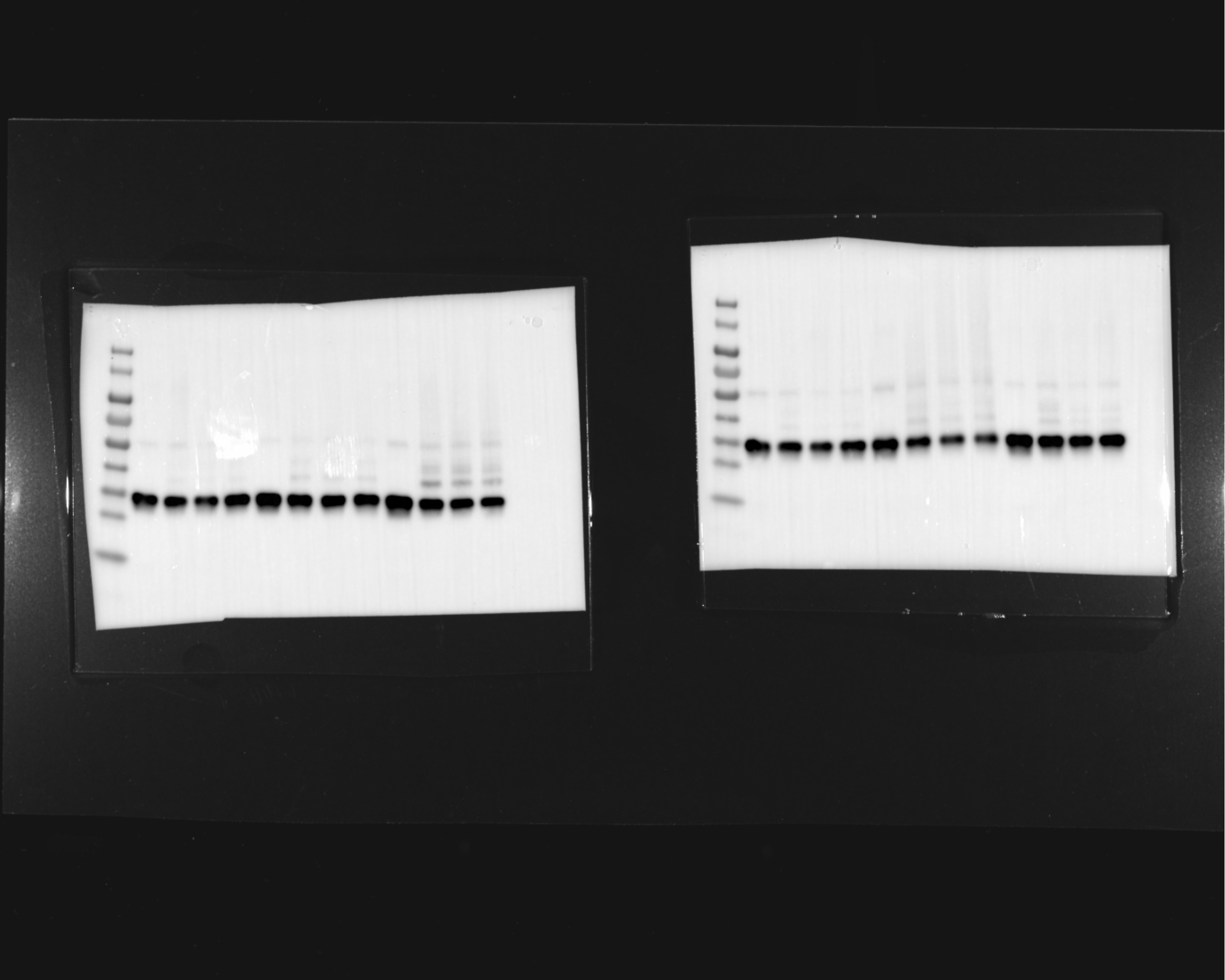

Supplement: Supplementary file 5 [file LSA-2021-01309_SdataS2.5.tif]

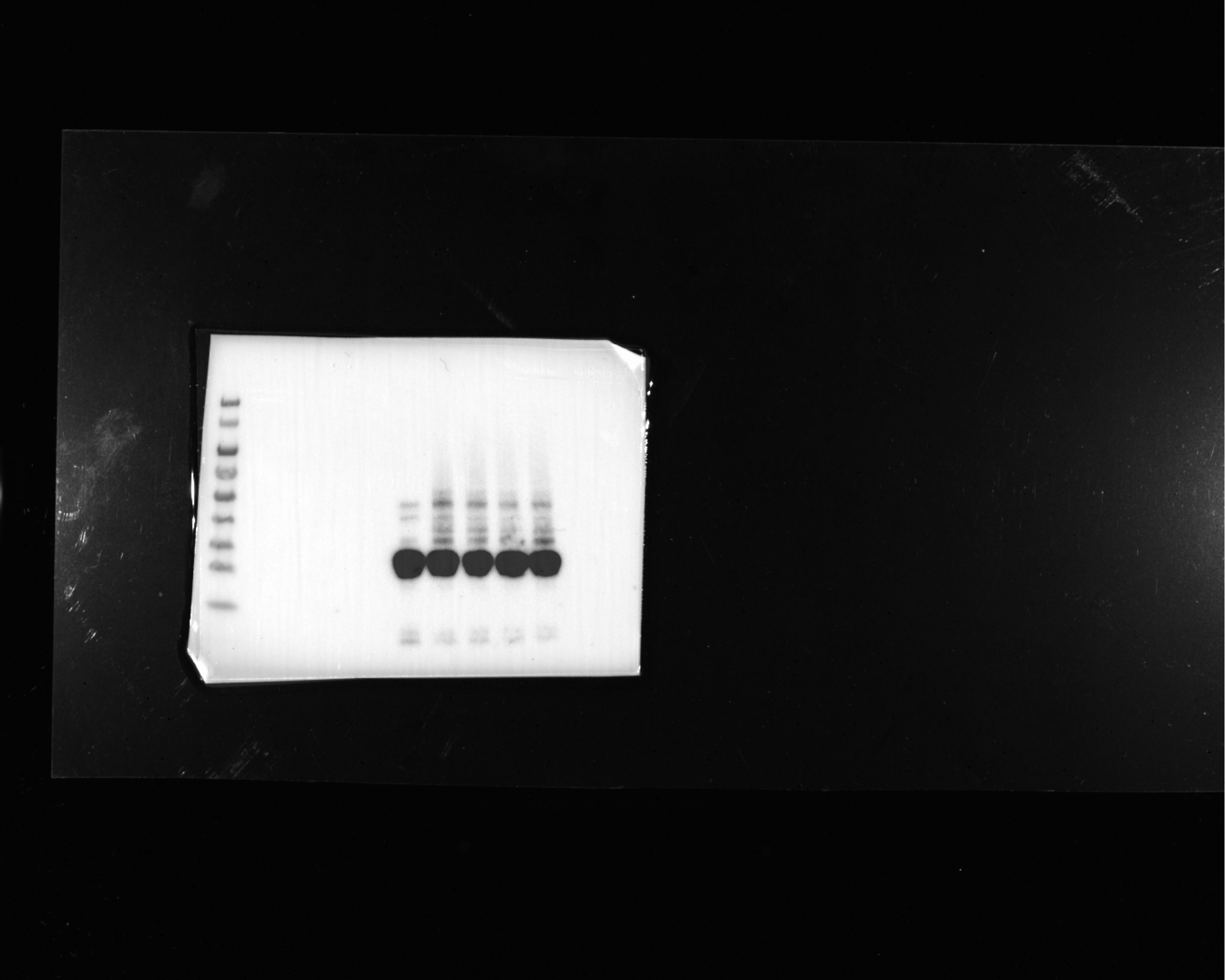

Supplement: Supplementary file 6 [file LSA-2021-01309_SdataS3.1.tif]

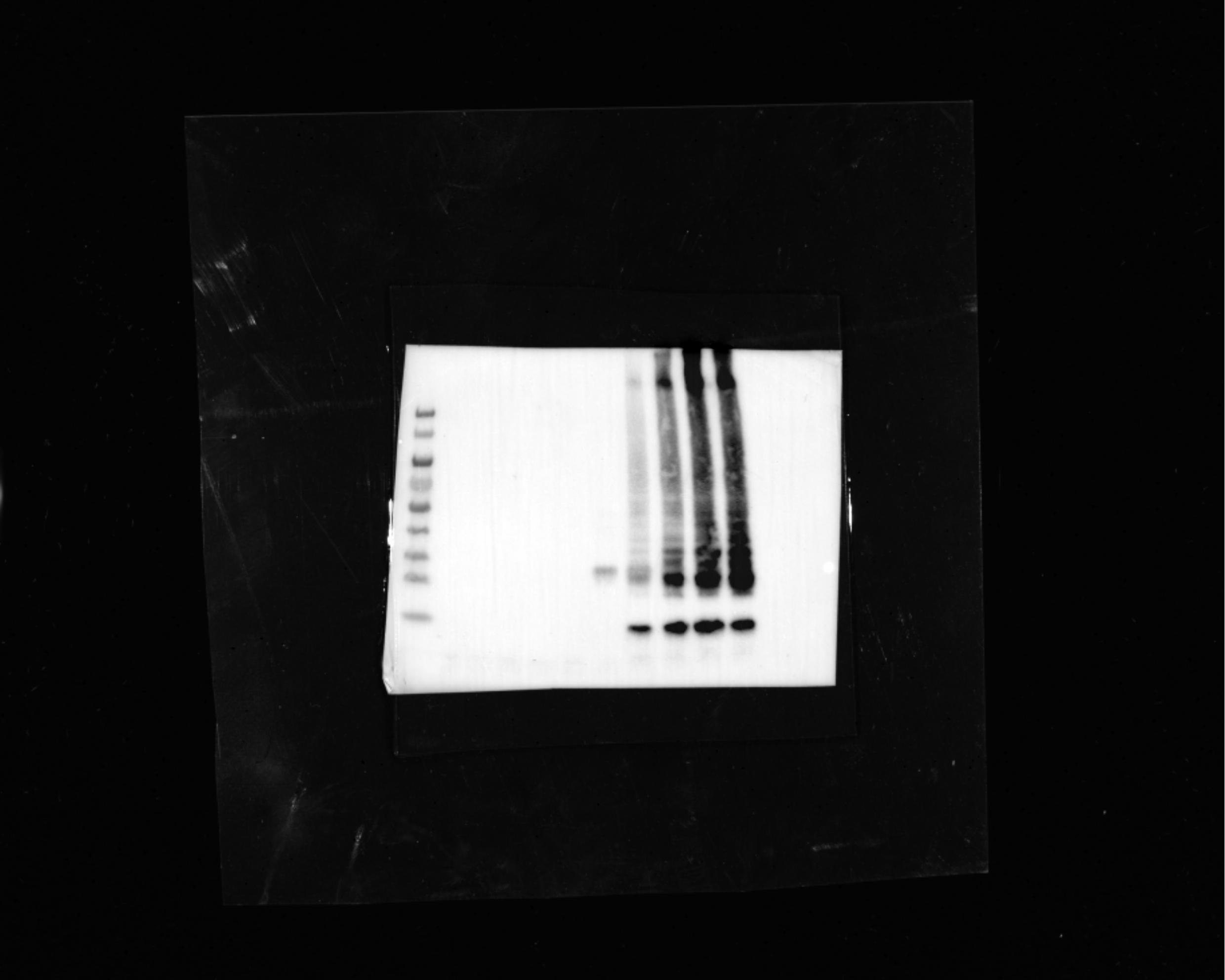

Supplement: Supplementary file 7 [file LSA-2021-01309_SdataS3.2.tif]

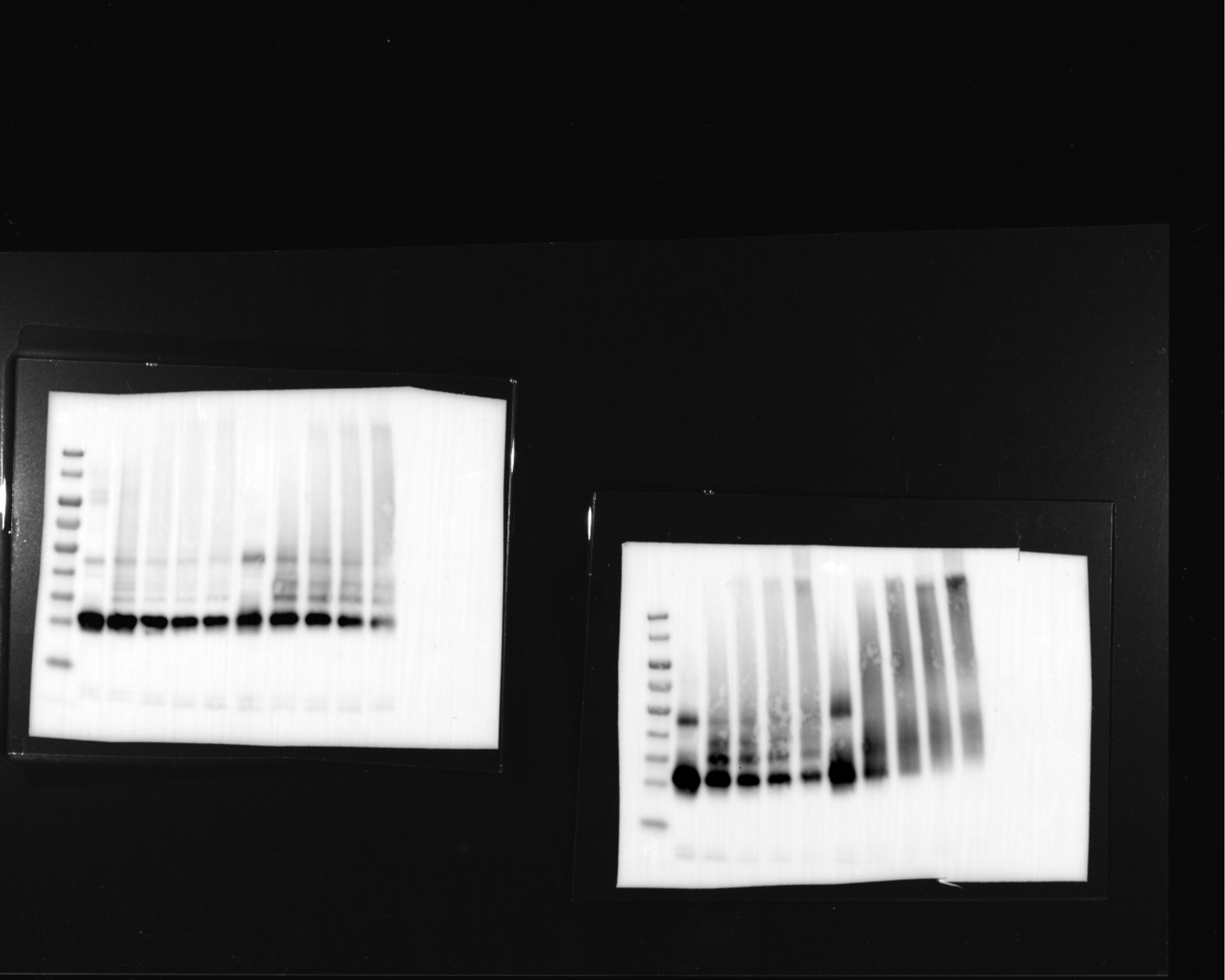

Supplement: Supplementary file 8 [file LSA-2021-01309_SdataS3.3.tif]

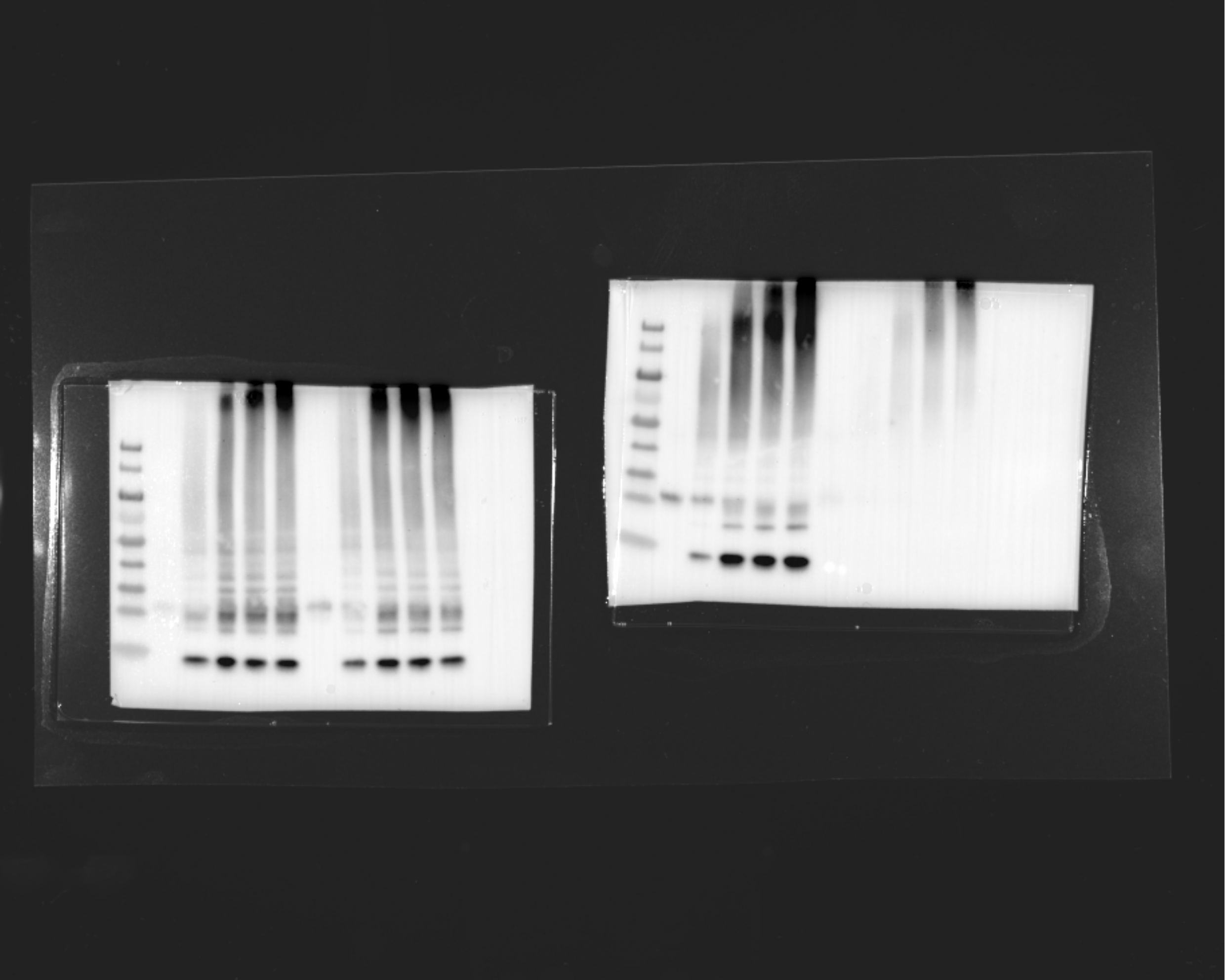

Supplement: Supplementary file 9 [file LSA-2021-01309_SdataS3.4.tif]

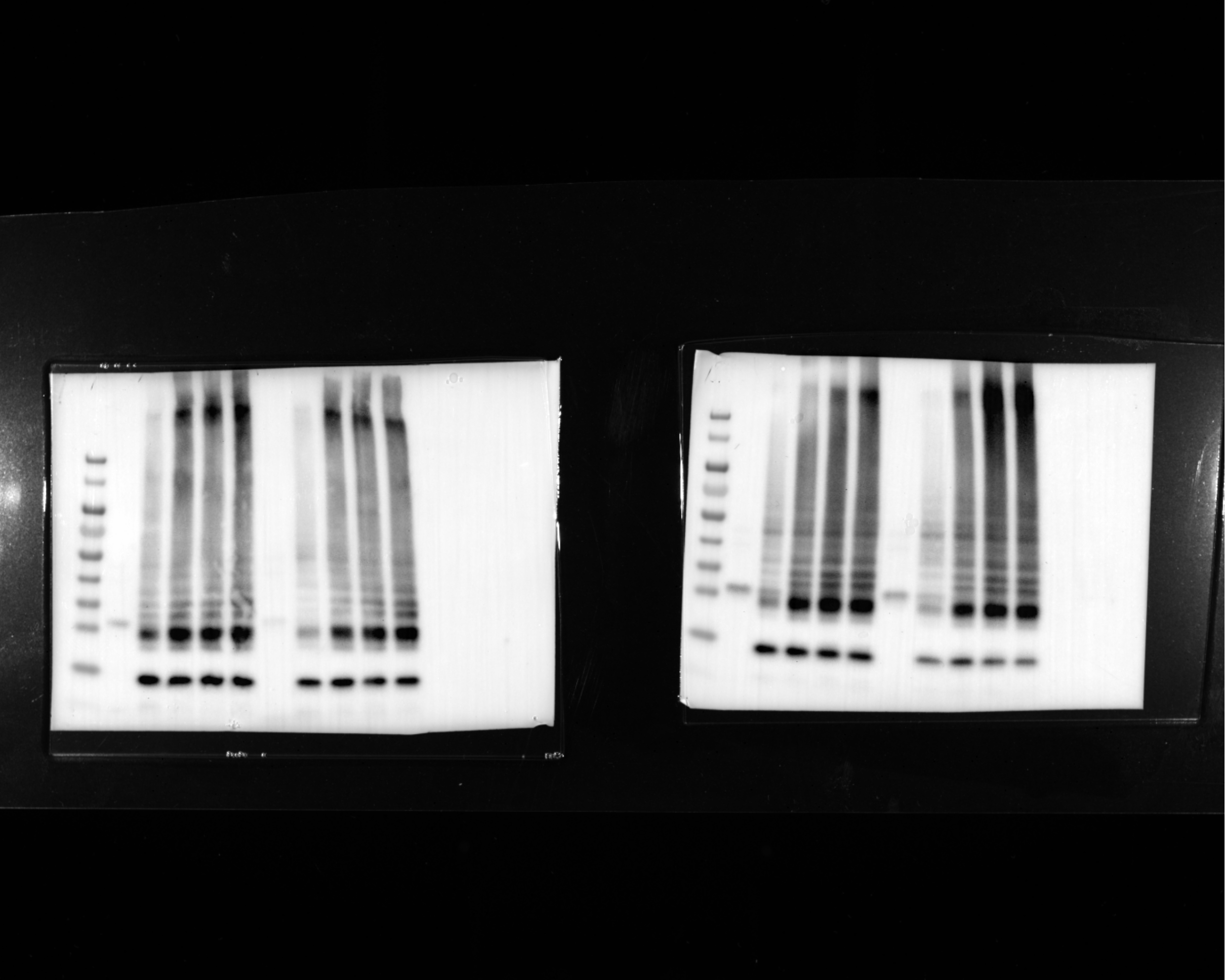

Supplement: Supplementary file 10 [file LSA-2021-01309_SdataS5.1.tif]

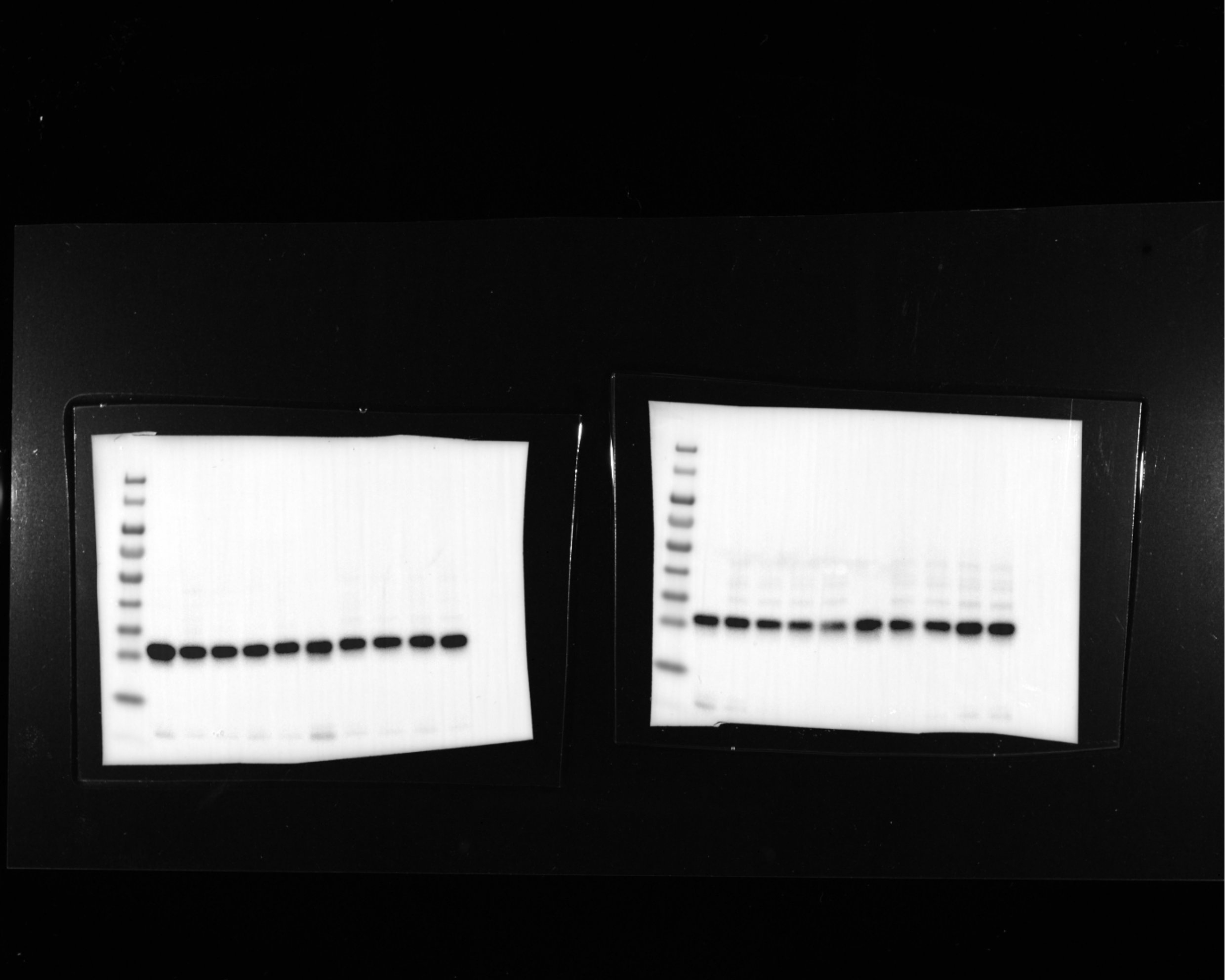

Supplement: Supplementary file 11 [file LSA-2021-01309_SdataS5.2.tif]

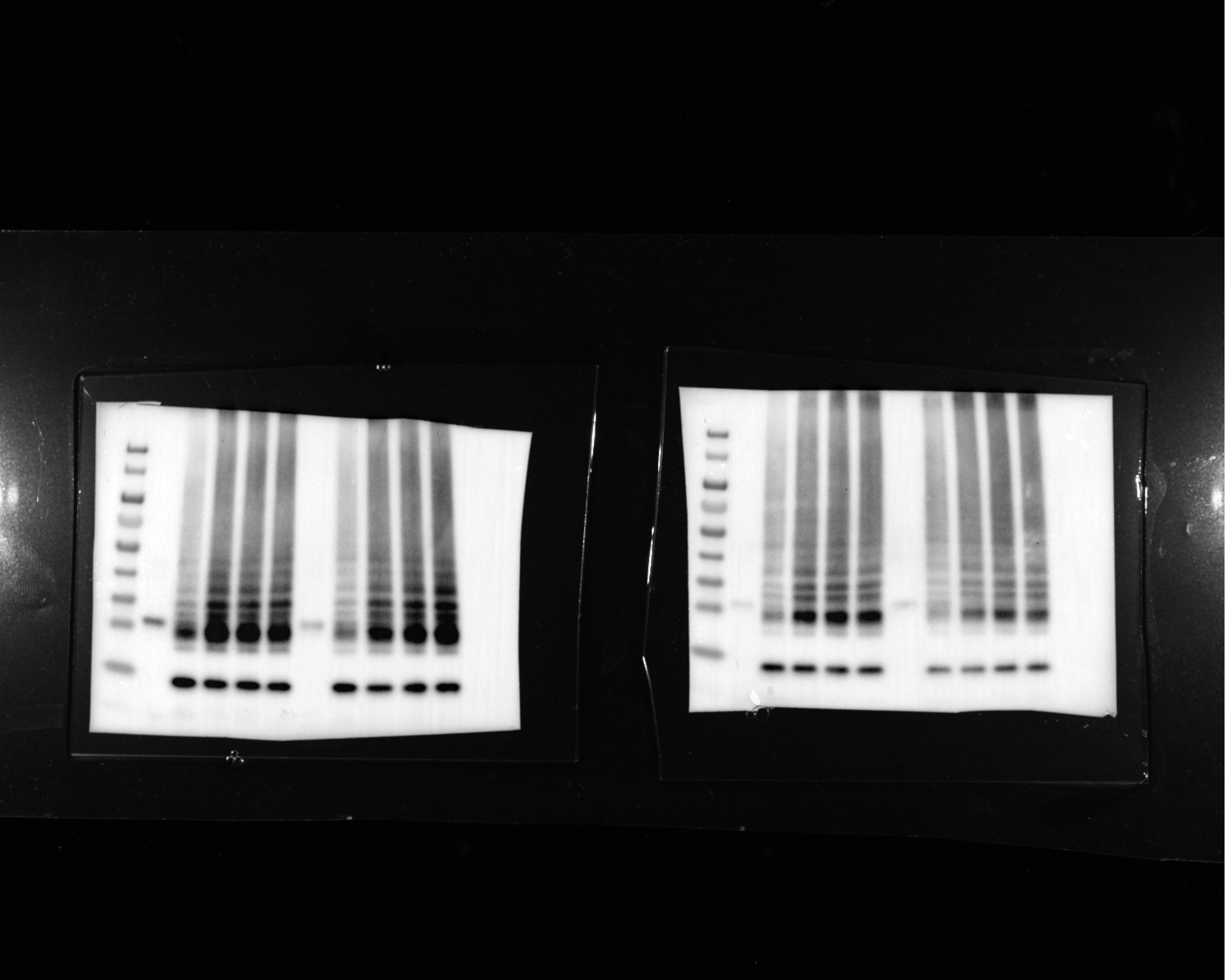

Supplement: Supplementary file 12 [file LSA-2021-01309_SdataS5.3.tif]

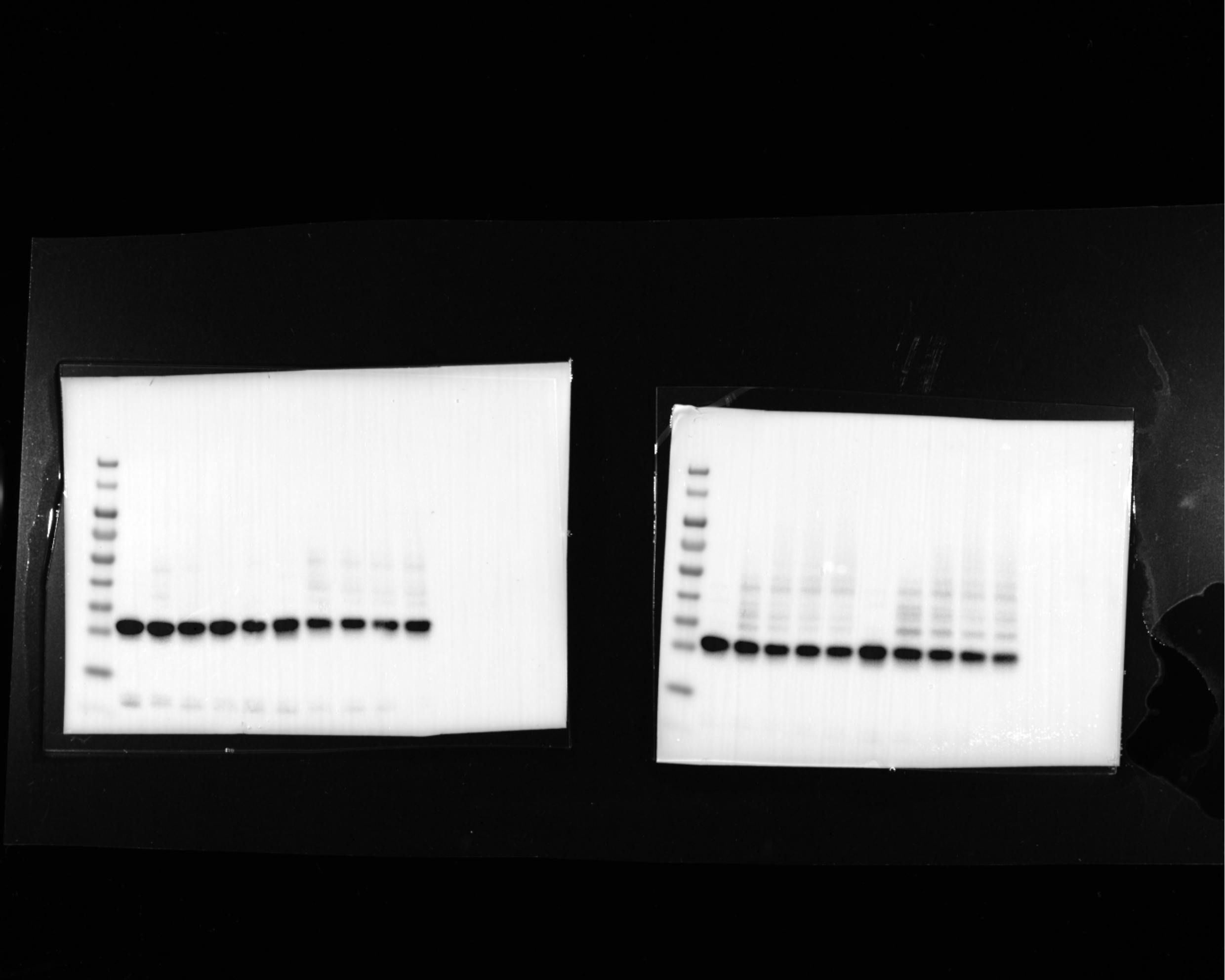

Supplement: Supplementary file 13 [file LSA-2021-01309_SdataS5.4.tif]
